# Marital Quality and Stress in Pregnancy Predict the Risk of Infectious Disease in the Offspring: The Norwegian Mother and Child Cohort Study

**DOI:** 10.1371/journal.pone.0137304

**Published:** 2015-09-30

**Authors:** Roger Ekeberg Henriksen, Frode Thuen

**Affiliations:** Centre for Evidence-Based Practice, Bergen University College, Bergen, Norway; University of Rennes-1, FRANCE

## Abstract

**Objectives:**

The aim of this study was to explore the degree to which couples’ relationship dissatisfaction and stressful life events during pregnancy predict the risk of infectious disease in the offspring during their first year of life.

**Methods:**

Data were obtained from the Norwegian Mother and Child Cohort Study, conducted by the Norwegian Institute of Public Health. Pregnant women completed questionnaires in week 30 of pregnancy concerning the couples’ relationship satisfaction and stressful life events. In follow-up questionnaires, the women reported whether their children (n = 74,801) had been subject to various categories of infectious disease: the common cold, throat infection, bronchitis, RS virus, pneumonia, pseudocroup, gastric flu, ear infection, conjunctivitis and urinary tract infection. Reports from two age groups of infants were used. Associations between the predictor and outcome variables were assessed via logistic regression and linear regression analyses.

**Results:**

Separate logistic regression analyses for each disease and age group showed that prenatal relationship dissatisfaction and stressful life events were significantly associated with all reported categories of infectious disease. After controlling for socioeconomic factors, social support, smoking, breastfeeding, maternal depression, the sex of the offspring, and use of child care, 29 out of 32 tested associations were statistically significant. Finally, multivariate linear regression analyses showed that prenatal relationship dissatisfaction and stressful life events were significantly associated with the frequency, as well as the variety, of infectious disease in the offspring.

## Introduction

Although child morbidity has been significantly reduced worldwide since the 1990s, infectious disease in infants remains a serious public health concern [1]. As it is the leading cause of hospitalization [2] and one of the main causes of death in infants [1,3], there is a strong need to identify factors that can reduce the likelihood of infant infections [1]. Moreover, early-life infections have been linked to long-term effects, such as adverse immune system development and an increased risk of asthma and allergies later in life [4,5]. The main purpose of this study was to examine to what degree the level of maternal relationship satisfaction during pregnancy predicts the risk of infectious disease in children during their first year of life. A second aim was to examine to what degree stressful prenatal life events predict the risk of infectious disease in the offspring.

Research has established that social relationships are essential for maintaining physical health [6]. In adulthood, romantic relationships have been recognized as being particularly important in predicting health outcomes [7–10]. It has, for instance, been shown that relationship quality predicts the risk of maternal infectious disease in pregnancy [11]. One likely explanation may be that individuals in low-quality relationships are more often subject to emotional distress related to spousal hostility and conflicts [12]. Hostile marital interactions and the physiological stress response that follows have, in turn, been linked to the impairment of the immune system [13]. Although no studies have yet examined whether maternal relationship quality during pregnancy is linked to the risk of infectious disease in the offspring, there is growing evidence from animal models that a link exists between prenatal maternal stress and a wide range of adverse health outcomes in the offspring, including immune dysfunction and infectious diseases [14,15]. Only a few studies on humans have investigated whether prenatal maternal stress and emotional distress compromise immune responses or predict infectious disease in the offspring [16–19]. The findings thus far are very much in line with findings in animal studies. For example, Nielsen and colleagues [17] found that in a Danish cohort study, women’s experiences of seriously stressful life events, such as the death of a spouse, the death of an older child, or divorce during pregnancy, were associated with a significantly higher risk of infections in their children. In a population-based cohort study, Tegethoff and colleagues [19] included measures of both emotional stress and stressful life events during pregnancy and found that both factors were associated with an increased risk of infectious disease in the offspring. In another study, Beijers and collegues [18] found that maternal prenatal anxiety and pregnancy specific difficulties, such as the fear of giving birth and the fear of bearing a child with disabilities, were related to infant respiratory infections, as well as to the use of antibiotics in the first year of life. A similar link may exist between prenatal maternal relationship dissatisfaction and the offspring’s risk of infectious diseases.

Although it remains unclear exactly how maternal experiences of stress during pregnancy affect the immune systems of infants, there is a consensus that the maternal hypothalamic-pituitary-adrenocortical (HPA) axis plays a key role [20]. The regulation of stress and negative emotions increases the release of corticotrophin-releasing hormone (CRH) from the hypothalamus, leading to the release of adrenocorticotrophic hormone (ACTH) from the pituitary gland [21,22]. ACTH travels to the adrenal cortex, where it stimulates the release of glucocorticoids, which in turn affect the immune system [23,24]. Animal studies have shown that maternal HPA axis activation not only affects the maternal immune system but also the offspring’s immune system [20,25,26]. Hence, any factor that may regulate HPA responses in pregnancy is interesting to investigate with regard to infant health. In the present study, our main interest is to investigate the potential impact of marital relationship quality during pregnancy. Several studies based on the general population show that marital distress may activate the HPA axis and predict adverse effects on maternal immune function [13,27,28]. Satisfaction with the partner, on the other hand, may reduce the stress-related activation of the HPA axis [29]. Similar research in the pregnant population is scarce. However, in a recent study, it was found that support from the partner down-regulated the effects of emotional distress on the release of cortisol in pregnant woman [30]. We therefore hypothesized that lower levels of prenatal maternal relationship satisfaction are associated with a higher risk of infectious disease in infants. A second aim of this study was to replicate the few existing studies in humans that investigate the relationship between stressful prenatal life events and the risk of infectious disease in the offspring. In order to test the hypotheses, we utilized data from the Norwegian Mother and Child Cohort Study.

## Materials and Methods

The Norwegian Mother and Child Cohort Study (MoBa) is a prospective population-based pregnancy cohort study conducted by the Norwegian Institute of Public Health. During the period from 1999 to 2008, all hospitals in Norway with more than 100 births per year, except two, invited pregnant women to participate in the study provided that they could read Norwegian [31]. They received a postal invitation three weeks before the ultrasound examination that was routinely offered to all pregnant women in Norway. Of the invited women, 40.6% consented to participate, and the cohort now includes 114,500 children, 95,200 mothers and 75,200 fathers. Blood samples were obtained from both parents during pregnancy and from mothers and children (umbilical cord) at birth. Follow-up was conducted via questionnaires at regular intervals and via linkage to national health registries. Several sub-studies were conducted to collect additional data and biological materials. The current study was based on Version 7 of the quality-assured data files released for research on 17 October 2012. Written informed consent was obtained from each MoBa participant upon recruitment. The study has obtained a license from the Norwegian Data Inspectorate and was approved by the Regional Committee for Medical Research Ethics.

### Participants

This study included married and cohabiting women participating in the Norwegian Mother and Child Cohort Study (n = 90,912) and their participating children (n = 100,027). Because we were interested in relationship quality, single and widowed mothers (and their children) were excluded. Some participants were also excluded from the multivariate analyses due to missing values for one or more of the research variables. Data from an average of 58,530 children were obtained for each category of infectious disease. It should be noted that the dataset contained very few data on the category ‘throat infection’ for 6- to 11 months old children. Thus, only 8,788 participants were included in the respective analysis. The participating women had a mean age of 29.6 years (standard deviation [SD] 4.55). In the general population in Norway, the mean maternal age at delivery was 29.5 between the years 2000–06 [32]. In the study population, 52% were married and 48% were cohabiting. In the total population of Norwegian women giving birth between the years 2000–06, 49% of all couples were married, and 43% were cohabiting [32]. In the study population, the average duration of the couples’ relationships was 6.3 years (SD 4.33). The average level of education was higher for the sample than for the general population of women aged between 16 and 49 years (official statistics from 2008 in brackets): compulsory school 7.0% (28.1%), vocational school 26.7% (35.7%), three-year college 39.2% (29.8%) and university/higher education 21.9% (6.4%). The full sample has been compared with the general population of pregnant women in Norway and is described in more detail elsewhere [32].

### Measures

Relationship satisfaction (RS) was measured during week 30 of the pregnancy using the full ten-item version of the relationship satisfaction scale. The scale was developed for the MoBa and is based on core items used in previously developed measures of marital satisfaction and relationship quality [33–35]. The scale has a six-step scoring format from 1 ‘totally agree’ to 6 ‘don’t agree at all’. Examples of items include the following: ‘My partner and I have problems in our relationship’, ‘I am very happy in my relationship’, ‘My partner is generally very understanding’ and ‘I am satisfied with my relationship with my partner.’ In the current sample, the scale had a Cronbach’s alpha of 0.90. This scale has been used in previous studies [11,36]. Prior to the logistic regression analyses and the linear regression analyses, the negative items were reverse coded so that all went in the same direction. As the highest score represents the lowest level of relationship satisfaction, this measure is referred to as ‘Relationship dissatisfaction’ in the tables and the results section.

Stressful life events (SLEs) were measured during week 30 of the current pregnancy and consisted of nine questions concerning different types of demanding experiences. The questions were inspired by the list in Coddington [37], and adapted to adult respondents. Participants were asked to report whether they had experienced any of the listed situations during the preceding 12 months. The questions include the following: ‘Have you had problems at work or where you study?’, ‘Have you had financial problems?’, ‘Have you been divorced, separated or ended your relationship with your partner?’, ‘Have you had problems or conflict with your family, friends, or neighbours?’, ‘Have you been seriously ill or injured?’, ‘Has anyone close to you been seriously ill or injured?’, ‘Have you been involved in a serious accident, fire or robbery?’, ‘Have you lost someone close to you?’, and ‘Other?’. The scoring format for each item was no (0) or yes (1). A scale was constructed based on sum scores. This scale reflects the number of stressful life events reported, ranging from 0 to 8. The scale has been used in a previous study [11].

Incidents of infectious diseases were recorded at six months after birth for the period when the child was less than six months old. Incidents of infectious diseases for the period when the child was between six and eleven months old were recorded eighteen months after birth. Due to the scope of the present study, only data from the first year of life was used. The recording was based on a checklist covering eight categories of infectious disease: the common cold, throat infection, pneumonia/RS-virus/bronchitis, diarrhea/gastric flu, ear infection, pseudocroup, urinary tract infection and conjunctivitis. The checklist was marked by the mothers, who were asked whether their children had or had not experienced a disease when the child was less than six months old and when it was between six and eleven months old. To measure the variety of diseases within each subject, a scale was constructed based on the number of categories of infectious diseases that were reported. To measure the frequency of diseases within each subject, another scale was constructed based on sum scores of how many times each disease had occurred during the given period of time.

### Control variables

Socioeconomic factors are possible confounding variables that are known to predict physical health outcomes and were therefore included as control variables. These variables included age, marital status (married versus cohabiting), household income (scored from 1 = no income to 7 = NOK 1,000,000 ≈ EUR 114,000) and educational level (six categories from public school to >4 years at university/college).

Smoking during pregnancy is associated with infant respiratory infections [38]. A dichotomized variable was constructed based on smoking sometimes, daily (= smoking) or not at all.

The breastfeeding of infants has been shown to reduce the risk of infectious disease in infancy [39]. For the purpose of the present study, a variable based on the sum scores for the number of months of breastfeeding was constructed.

Social support may buffer stress-related HPA responses [40], and the adverse consequences of poor social relationships on immune-functioning have been well-documented [41]. To control for this, we measured the frequency of contact with family and close friends, as well as the possibility of seeking advice from people other than the husband/partner in a difficult situation. A scale with a three-step scoring format was constructed based on the following questions: ‘Do you have anyone other than your husband/partner you can ask for advice in a difficult situation?’ and ‘How frequently do you meet or talk on the telephone with your family (other than your husband/partner and children) or close friends?’

The sex of the offspring was included as a possible confounding factor because prenatal stress has been shown to have sex-specific effects on the developing fetus [15].

Maternal depression and relationship dissatisfaction frequently co-occur [42]. Moreover, maternal depression may have adverse effects on the fetal development [43]. Maternal depression was therefore included as a possible confounding factor. Depression was measured based on one item from a checklist covering a wide range of diseases and health problems. The respondents were asked to mark whether they had or had had ongoing depression between the following weeks of the current pregnancy: 13–16, 17–20, 21–24, 25–28, 29+. A variable was constructed based on the mean scores of each time interval (scoring format: no depression = 0, depression = 1).

Finally, the use of kindergarten or other child care facilities was included as a control variable because child care is associated with higher exposure to virus and bacteria [44]. Because child care facilities were not used for < 6-month-old infants, this variable was only included in analyses based on the group of 6- to 11-month-old infants.

### Statistical analyses

All statistical analyses were conducted using IBM SPSS, Version 22. Descriptive analyses were conducted to examine the nature of the study variables. Because maternal antibodies (IgG) may protect the offspring during the first 6 months of life [45], the data for the two age groups of infants were analyzed separately. The associations between the level of maternal relationship satisfaction and infectious disease in the group of < 6-month-old infants were first tested by performing separate bivariate logistic regression analyses for each of the eight infectious diseases as the dependent variable, using the level of relationship satisfaction as the predictor variable. This procedure was repeated for the group of 6- to 11-month-old infants. In order to assess the unique contribution of the level of relationship satisfaction, multivariable logistic regression analyses were performed with the following independent control variables: stressful life events, maternal age, level of education, income, marital status, social support, breastfeeding, smoking during pregnancy, maternal depression and the sex of the offspring. For the analyses based on the group of 6- to 11-month-old infants, use of child care was also included as a control variable. The same procedure was used to test the associations between prenatal stressful life events and infectious diseases in the offspring, except that maternal relationship satisfaction was included as a control variable in the multivariable analyses. Multiple linear regression analyses were conducted to examine the associations between prenatal relationship satisfaction and stressful life events, and the frequency of reported episodes of infectious disease in the offspring. Finally, multiple linear regression analyses were conducted to examine the associations between prenatal relationship satisfaction and stressful life events, and the variety of infectious diseases in the offspring. The analyses were conducted separately for each of the two age groups of infants: less than 6 months old and 6- to 11 months old.

## Results

The descriptive analyses showed that at least one infectious disease was reported in 99.9% of the infants in both age groups. The mean number of types of infectious diseases was 1.21 (SD = 0.92) in the youngest infants (< 6 months old) and 1.71 (SD = 2.68) in the oldest infants (6- to 11 months old). The mean number of reported infectious disease episodes (across all types) was 2.66 (SD = 2.48) in the youngest infants (< 6 months old) and 3.34 (SD = 2.68) in the oldest infants (6- to 11 months old). The incidences of each disease are displayed in Table 1 for the youngest infants and Table 2 for the oldest infants.

**Table 1:** Associations between stressful life events and relationship dissatisfaction in pregnancy, and infectious diseases in the less-than-6-month-old children.

Category of infection	Incidence	N	Stressful life events			Relationship dissatisfaction,		
	%		unadjusted OR	adjusted OR^1^	95% CI^1^	unadjusted OR	adjusted OR^2^	95% CI^2^
Common cold	76.7	68,168	1.06*	1.05*	1.04–1.07	1.25*	1.31*	1.27–1.35
Throat infection	3.0	67,123	1.16*	1.14*	1.09–1.18	1.30*	1.25*	1.17–1.34
Ear infection	4.6	67,124	1.08*	1.05*	1.02–1.09	1.24*	1.23*	1.16–1.30
Pseudocroup	2.2	67,109	1.07*	1.06*	1.01–1.12	1.14*	1.14*	1.05–1.24
Bronchitis /RS virus/ Pneumonia	4.9	67,173	1.04*	1.02	0.99–1.05	1.24*	1.26*	1.19–1.33
Gastric flu / diarrhea	11.3	67,165	1.13*	1.13*	1.10–1.15	1.12*	1.11*	1.07–1.15
Urinary tract infection	0.9	67,067	1.11*	1.08*	1.00–1.17	1.15*	1.17*	1.03–1.34
Conjunctivitis	25.9	67,247	1.05*	1.05*	1.03–1.07	1.19*	1.21*	1.17–1.24

^1^ Odds ratio adjusted for relationship dissatisfaction, age, level of education, income, marital status, social support other than partner, smoking, maternal depression, breastfeeding and the sex of the offspring

^2^ Odds ratio adjusted for stressful life events, age, level of education, income, marital status, social support other than partner, smoking, maternal depression, breastfeeding and the sex of the offspring

* Unadjusted Odds ratio = p < 0.05

**Table 2:** Associations between stressful life events and relationship dissatisfaction in pregnancy, and infectious diseases in the 6- to 11-month-old children.

Category of infection	Incidence	N	Stressful life events			Relationship dissatisfaction,		
	%		unadjusted OR	adjusted OR^1^	95% CI^1^	unadjusted OR	adjusted OR^2^	95% CI^2^
Common cold	87.8	56,457	1.04*	1.03*	1.00–1.05	1.18*	1.23*	1.18–1.29
Throat infection	7.5	8,788	1.11*	1.09*	1.02–1.18	1.18*	1.18*	1.03–1.34
Ear infection	15.5	55,478	1.09*	1.07*	1.05–1.09	1.16*	1.16*	1.11–1.20
Pseudocroup	6.1	56,218	1.07*	1.07*	1.03–1.11	1.08*	1.10*	1.04–1.17
Bronchitis /RS virus/ Pneumonia	6.2	55,956	1.08*	1.06*	1.02–1.09	1.17*	1.19*	1.12–1.26
Gastric flu / diarrhea	31.6	54,028	1.08*	1.07*	1.06–1.09	1.15*	1.16*	1.12–1.19
Urinary tract infection	1.5	56,452	1.09*	1.06	0.99–1.13	1.09°	1.09	0.98–1.22
Conjunctivitis	25.3	55,020	1.05*	1.04*	1.02–1.06	1.18*	1.21*	1.17–1.25

^1^ Odds ratio adjusted for relationship dissatisfaction, age, level of education, income, marital status, social support other than partner, smoking, maternal depression, breastfeeding, the sex of the offspring, and use of childcare.

^2^ Odds ratio adjusted for stressful life events, age, level of education, income, marital status, social support other than partner, smoking, maternal depression, breastfeeding, the sex of the offspring, and use of childcare.

* Unadjusted Odds ratio = p < 0.05

° Unadjusted Odds ratio = p = 0.121

The majority of pregnant women reported low levels of relationship dissatisfaction (M = 1.65, SD = 0.65). At least one stressful life event (SLE) within the 12 months before gestational week 30 was experienced by 56% of the pregnant women (one SLE: 27.9%; two SLEs: 15.8%; three SLEs: 6.1%; four SLEs: 2.0%. Only 0.6% of the participants reported five or more SLEs). The mean number of reported stressful life events was 0.95 (SD = 1.09) and ranged from 0 to 8. Within the group of young infants, the bivariate logistic regression analyses showed a significant association between maternal relationship dissatisfaction and all eight infectious diseases (Table 1). Among the older infants, all diseases except one were significantly related to relationship dissatisfaction (Table 2). The associations remained significant within a 95% level of confidence for both age groups after adjusting for scores regarding prenatal stressful life events, age, education, income, marital status, social support, smoking, maternal depression, breastfeeding, and the sex of the offspring. For the 6- to 11-month-old children, the use of child-care was also included as a control variable. Bivariate and multivariate logistic regression analyses were performed to test the associations between stressful prenatal life events and infectious diseases. As shown in Tables 1 and 2, the results from the bivariate logistic regression analyses showed a significant association with all eight infectious diseases in both age groups of infants. After adjusting for scores regarding prenatal relationship dissatisfaction, age, education, income, marital status, social support, smoking, breastfeeding, maternal depression, the sex of the offspring and the use of child-care (for the 6- to 11-month-old), the statistical significance of the associations persisted for seven out of eight diseases in the youngest group (Table 1), and for seven out of eight diseases in the oldest group (Table 2).

Multiple linear regression analyses were conducted to examine the association between relationship dissatisfaction, stressful life events and the frequency and variety of infectious diseases reported. The two main predictor variables and the control variables were entered simultaneously. The control variables were the same as used before on the two groups of children. Two models were constructed to examine the association between relationship dissatisfaction, stressful life events and the frequency of reported episodes of infectious diseases within the two age groups of infants: less than 6 months old and 6- to 11 months old. After the inclusion of the control variables, the model of the youngest group explained 0.9% of the variance (adjusted R^2^ = .009, F(11,55649) = 48.048, p < .001). The model of the oldest group explained 1.6% of the variance (adjusted R^2^ = .016, F(12,50868) = 71.563, p < .001). The results showed that both main predictor variables were significantly associated with the frequency of infectious diseases in the offspring. The results are displayed in Table 3. Two additional models were constructed to examine the associations between relationship dissatisfaction, stressful life events and the number of various infectious diseases in the offspring. After the inclusion of the control variables, the model of the less-than-6-month-old infants explained 1.7% of the variance (adjusted R^2^ = .017, F(11,68578) = 108.981, p < .001). The model of the 6- to 11 month-old infants explained 1.8% of the variance (adjusted R^2^ = .018, F(12,57353) = 88.271, p < .001). As shown in Table 3, prenatal relationship dissatisfaction, as well as stressful life events, were significantly associated with the number of infectious diseases in the offspring.

**Table 3:** Unstandardised and standardised regression coefficients for relationship dissatisfaction and stressful life events in pregnancy on number of infectious diseases in the offspring.

	Frequency of infections^1^					Variety of infections^2^				
**Less-than-6-month-old infants (n = 55,660)**	B	SE B	β	T	p	B	SE B	β	t	p
Relationship dissatisfaction	.263ᵃ	.018	.064ᵃ	14.623	< .001	.124ᵃ	.006	.081ᵃ	20.479	< .001
Stressful life events	.092ᵇ	.010	.039ᵇ	9.118	< .001	.041ᵇ	.003	.047ᵇ	12.124	< .001
**6- to 11-month-old infants (n = 50,880)**										
Relationship dissatisfaction	.280ᶜ	.020	.063ᶜ	13.690	< .001	.125ᶜ	.008	.069ᶜ	16.114	<. 001
Stressful life events	.105ᵈ	.011	.041ᵈ	9.230	< .001	.043ᵈ	.004	.042ᵈ	10.004	< .001

^1^ Frequency = the number of reported episodes of infections across the eight categories of diseases.

^2^ Variety = the number of categories of infectious diseases that were reported.

^a^ Adjusted for scores on stressful life events, age, level of education, income, marital status, social support, smoking, maternal depression, breastfeeding and sex of the offspring.

^b^ Adjusted for scores on relationship dissatisfaction, age, level of education, income, marital status, social support, smoking, maternal depression, breastfeeding and sex of the offspring.

^c^ Adjusted for scores on stressful life events, age, level of education, income, marital status, social support, smoking, maternal depression, breastfeeding, sex of the offspring and use of childcare.

^d^ Adjusted for scores on relationship dissatisfaction, age, level of education, income, marital status, social support, smoking, maternal depression, breastfeeding, sex of the offspring and use of childcare.

## Discussion

This study examined the degree to which prenatal maternal relationship dissatisfaction and stressful life events predict the risk of infectious disease in the offspring in their first year of life. The study was based on a nationwide Norwegian pregnancy cohort. Data on four groups of respiratory tract infections and four other groups of infections were included in the linear and logistic regression analyses. We found that maternal relationship dissatisfaction was significantly associated with all eight infectious diseases among the less-than-6-month-old infants. Within the group of 6- to 11-month-old infants, maternal relationship dissatisfaction was associated with seven out of eight infection categories. The only exception was urinary tract infection. We also found that women who reported higher degrees of relationship dissatisfaction during pregnancy reported both a higher frequency, as well as a higher variety, of infectious diseases in their offspring. The associations were generally weak but consistent across the age groups. This suggests that relationship dissatisfaction was not only associated with reoccurring symptoms of a specific infectious disease but also with a general susceptibility to various infectious diseases.

The present results contribute to new knowledge suggesting that marital dissatisfaction in pregnancy is a factor that affects the risk of infectious diseases in infants. On average, for each one-point increase in relationship dissatisfaction, the odds of reporting one infectious disease increased by a factor of 1.21. This means that the children of mothers who reported the highest possible scores on relationship dissatisfaction during pregnancy had, on average, 2.6 times higher odds of suffering from an infectious disease as compared with the children of mothers who reported the lowest possible scores. We were not able to find any previous studies that had focused on couples’ relationship quality during pregnancy and its impact on the occurrence of infectious disease in the offspring. It is interesting to note, however, that the magnitude of the associations in this study were similar to those found in a recent study that examined the associations between maternal relationship satisfaction in early pregnancy and self-reported infectious diseases later in pregnancy [11].

The associations between stressful life events and infections followed a similar pattern as the associations between relationship dissatisfaction and infections. The logistic regression analyses showed that stressful prenatal life events were significantly associated with seven out of eight categories of infectious diseases in the young infants. The exception was for the bronchitis/RS virus/pneumonia category. Among the oldest infants, stressful life events were also significantly associated with seven out of eight categories of disease. The exception was urinary tract infection. As with lower marital satisfaction, based on linear regression analyses, the association between stressful life events and infections showed that prenatal stressful life events predicted both higher frequency and a greater variety of infectious diseases during the first year of life. These findings are generally in line with previous studies showing that stressful prenatal life events predict infectious disease in infants, as well as in older children [17–19]. Interestingly, we found that the associations were stronger for marital dissatisfaction than for stressful life events. The difference may be due to the periodic nature of stressful life events versus the chronicity of marital difficulties. A measure of daily stress may be more appropriate for a comparison with the effects of relationship dissatisfaction.

The present study did not provide data about underlying physiological mechanisms. However, one of the major proposed mechanisms to account for the relationship between prenatal psychosocial stress and the offspring’s susceptibility to infections is the secretion of cortisol, which is regulated by the maternal HPA axis and the placenta [14,20]. Both animal and human studies suggest that stress-related maternal cortisol increases the fetus’s exposure of cortisol and subsequently affect the development of the fetus’s immune domains [26,46]. Very few studies, however, have investigated the link between maternal cortisol and infectious diseases in infants [47]. One exception is the work of Beijers and colleagues [18] who found that higher evening cortisol levels and flattened diurnal cortisol rhythms in late pregnancy were related to increased infant respiratory infections. They also found a significant correlation between pregnancy-specific distress and evening cortisol levels. Indeed, other researchers have demonstrated that both stressful life events and partner support can regulate cortisol levels in pregnant women [30,48,49]. Thus, the physiological mechanisms underlying the associations found in the present study may be linked to maternal HPA axis activation.

The causal pathways described above may apply for relationship dissatisfaction, as well as for stressful life events. However, there are other possible explanations for the findings. For instance, it is possible that stress-related emotional distress and maternal relationship dissatisfaction persist after delivery. This could provide a stressful environment for the offspring by affecting mother-child interactions and infant stress reactivity [50]. The infant may respond with the heightened secretion of glucocorticoids, leading to adverse immune-regulation and an increased risk of infections as a consequence. Unfortunately, the dataset did not allow us to control for this possibility. A comprehensive review of additional potential mechanisms is provided by Beijers and colleagues [20].

The present study utilized a large amount of data from a large number of participants. This allowed for assessing relatively small effect sizes within a narrow confidence interval. At any rate, the results must be interpreted with some caution. One concern is that the study relied on self-report questionnaires. It is, for example, possible that the validity and reliability of the outcome measure were weakened by inaccurate interpretations of the children’s symptoms. Another limitation was that bronchitis, RS virus and pneumonia were collapsed into one category. Moreover, we were unable to control the timing of the impact of the predictor variables. For instance, we do not know whether the stressful life events occurred before or during pregnancy. This was due to the design of the questionnaire. Also, the data did not allow for evaluating whether the effects of stressful life events and relationship dissatisfaction were related to chronic stress or time-limited distress. It has been suggested that the timing may be significant because the development of the fetal immune system is affected differently depending on gestational stage [25]. Another limitation was that the data did not allow for controlling for the effect of other children in the household. Having a child is linked with poorer relationship satisfaction [51], and is also naturally linked with whether or not the baby is exposed to illnesses from a sibling. Thus, the number of children in the household may be a confounding variable. It should also be mentioned that only 40% of the invited women consented to participate. However, the potential impact of selection bias on exposure and outcome variables was evaluated in a study that tested eight different exposure—outcome associations [32]. The authors found no statistically significant differences in association measures between participants in the Norwegian Mother and Child Cohort Study and the total population, indicating that the generalizability of this study is not violated by selection bias. Finally, it is a concern that the scores for the main predictor variables were not normally distributed. In small samples, this may lead to problems with the interpretation of tests of statistical significance. However, in large sample sizes, the non-normality of the residuals will not hamper the interpretation of confidence intervals [52].

To conclude, our study provides empirical evidence that links stressful prenatal life events with the risk of infectious diseases in the infant offspring. Moreover, we found that lower levels of relationship satisfaction during pregnancy increased the risk, as well as the frequency and variety, of infectious diseases in infants. This may have implications for public health policy. However, statistically significant findings in large samples does not necessarily yield clinical significance. It remains to examine whether relationship management before or during pregnancy may reduce the risk of diseases in the offspring. Future studies should aim to increase our understanding of how and to what degree the prenatal social environment influences the physical health of the offspring over their lifespans.
